# AMPA Receptors Exist in Tunable Mobile and Immobile Synaptic Fractions *In Vivo*

**DOI:** 10.1523/ENEURO.0015-21.2021

**Published:** 2021-05-14

**Authors:** Haiwen Chen, Richard H. Roth, Elena Lopez-Ortega, Han L. Tan, Richard L. Huganir

**Affiliations:** 1 Division of Pediatric Neurology, Department of Neurology, Johns Hopkins University School of Medicine, MD 21205; 2 Department of Pediatrics, Johns Hopkins University School of Medicine, MD 21205; 3 Solomon H. Snyder Department of Neuroscience and Kavli Neuroscience Discovery Institute, Johns Hopkins University School of Medicine, Baltimore, MD 21205

**Keywords:** GluA1, plasticity, stress, synapses

## Abstract

AMPA receptor (AMPAR) mobility within synapses has been extensively studied *in vitro*. However, whether similar mobility properties apply to AMPARs *in vivo* has yet to be determined. Here, we use two-photon fluorescence recovery after photobleaching (FRAP) to study AMPAR mobility within individual dendritic spines in live animals using an overexpression vector. We demonstrate the existence of mobile and immobile fractions of AMPARs across multiple cortical regions and layers. Additionally, we find that AMPAR mobility can be altered *in vivo* in response to administration of corticosterone, a condition that mimics exposure to stress.

## Significance Statement

Our work provides novel insight into receptor mobility within intact brains of live mice using two-photon microscopy through cranial windows. *In vivo* assessment of protein mobility within mammalian neuronal synapses has thus far been limited. Here, within this system, we are able to confirm that there are both mobile and immobile AMPA receptor (AMPAR) fractions *in vivo* and that these fractions are similar across different cortical regions and layers. Additionally, we reveal that the proportion of mobile to immobile receptor fraction may be altered by administration of corticosterone, a condition that mimics stress response, suggesting AMPAR mobility is acutely modulated *in vivo*.

## Introduction

Trafficking of AMPA receptors (AMPARs) modulates synaptic strength at excitatory synapses. This process underlies the plasticity of neurons that supports learning and memory (Huganir and Nicoll, 2013; Diering and Huganir, 2018). In postsynaptic spines, AMPARs laterally diffuse along the plasma membrane and may enter and exit synapses (Borgdorff and Choquet, 2002; Heine et al., 2008). Within synapses themselves, a combination of protein interactions and molecular crowding constrains and immobilizes a large fraction of AMPARs (Bats et al., 2007; Li et al., 2016). The clustered distributions of these synaptic AMPARs and their relative position to presynaptic vesicle release machinery further regulate the efficacy of synaptic transmission (MacGillavry et al., 2013; Nair et al., 2013; Tang et al., 2016).

AMPAR mobility has previously been assessed within individual synapses *in vitro*. One technique utilizes fluorescence recovery after photobleaching (FRAP) of fluorescently tagged AMPARs, whereby fluorescence recovery reflects exchange of bleached molecules in the spine with unbleached molecules elsewhere in the cell, representing a mobile fraction of molecules. Conversely, a lack of fluorescence recovery reflects the immobile protein fraction.

Within dissociated hippocampal cell cultures, FRAP of fluorescently tagged AMPAR subunit GluA1 revealed a 56% mobile fraction within dendritic spines (Sharma et al., 2006). Other such studies provided a range between 20% and 60% (Ashby et al., 2006; Bats et al., 2007; Heine et al., 2008; Frischknecht et al., 2009; Martin et al., 2009; Arendt et al., 2010). Using super-resolution single molecule tracking, the mobile fraction of GluA1 was estimated to be ∼70%, where half of these receptors exchanged between synaptic and extrasynaptic domains and the other half was mobile within the synapse (Heine et al., 2008). In contrast, within organotypic hippocampal cultures, Makino and Malinow (2009) found 103% recovery of GluA1 with FRAP at basal states, suggesting that GluA1-containing AMPARs were entirely mobile. They proposed that this is because recombinant GluA1 remains within spine surface pools rather than being stably incorporated into synapses at basal states. They further observed a decrease in GluA1 FRAP recovery to ∼70% only with induction of chemical long-term potentiation (LTP), suggesting a portion of those receptors had been incorporated into the immobile synaptic fraction with stimulation, consistent with previous experiments on LTP in organotypic cultures (Hayashi et al., 2000; Kopec et al., 2006). In further contrast, within acute slices, FRAP of chemically labeled endogenous GluA1 was reported to recover only ∼10% fluorescence (Wakayama et al., 2017). Notably, there is overall significant disparity in the characterization of AMPAR dynamics between different experimental systems, in part because of differences between *in vitro* conditions. Which system most accurately reflects biology *in vivo* is unknown.

Therefore, we sought to assess AMPAR mobility at individual spines of cortical neurons in intact brains of live animals employing *in vivo* two-photon FRAP of fluorescently labeled AMPARs.

## Materials and Methods

### Animals

Experiments were performed using male and female adult (two to four months) wild-type (WT) C57BL/6N mice (Charles River). All animals were treated in accordance with the Johns Hopkins University Animal Care and Use Committee guidelines. Animals were kept on a 12/12 h light/dark cycle.

### *In utero* electroporation and cranial window implantation

*In utero* electroporation and cranial window implantation were performed as previously described (Zhang et al., 2015; Roth et al., 2020; Tan et al., 2020). Briefly, *in utero* electroporation of SEP-GluA1, myc-GluA2, and DsRed2 at a 4:2:1 ratio was performed on embryonic day (E)15 embryos from timed pregnant C57BL/6N mice in L2/3 of visual cortex or E13 embryos for L5 of either motor or visual cortices. Pups born after, both males and females, were implanted with a cranial window overlying respective transfected cortices at 8–12 weeks of age, and a custom-made metal head bar was attached to the skull to fixate the mouse for imaging.

### Two-photon FRAP imaging

*In vivo* two-photon imaging was performed under isoflurane anesthesia (0.5% volume isoflurane/volume O_2_) using a Zeiss two-photon laser-scanning microscope with a 20 × 1.0 NA water immersion objective lens (Zeiss). SEP-GluA1 and DsRed2 were excited at 910 nm with a Ti:sapphire laser (Coherent) with ∼100-mW power delivered to the back-aperture of the objective. Image stacks of 12–14 z-steps were acquired at 512 × 512 pixels with pixel size of 0.21 μm in *x* and *y* and z-steps of 1 μm with pixel dwell times of 2 μs/pixel. Baseline image stacks were acquired 5 min apart before bleaching and FRAP image stacks acquired immediately after photobleaching and at times indicated in the figures. Photobleaching of spines were achieved with repetitive xy scanning of select 10 × 10-pixel regions of interest (ROIs) at the center plane of the image stack at high illumination intensity at 910 nm for dwell times of 32–65 μs/pixel with one to two iterations. For each experiment, ∼10–15 spines were bleached at a time for total bleach times of 30–60 s. Spines targeted for photobleaching resulted in ∼50% reduction of fluorescence in SEP-GluA1 and ∼15% decrease in fluorescence in DsRed2 (Extended Data Fig. 1-1*D*).

**Figure 1.**
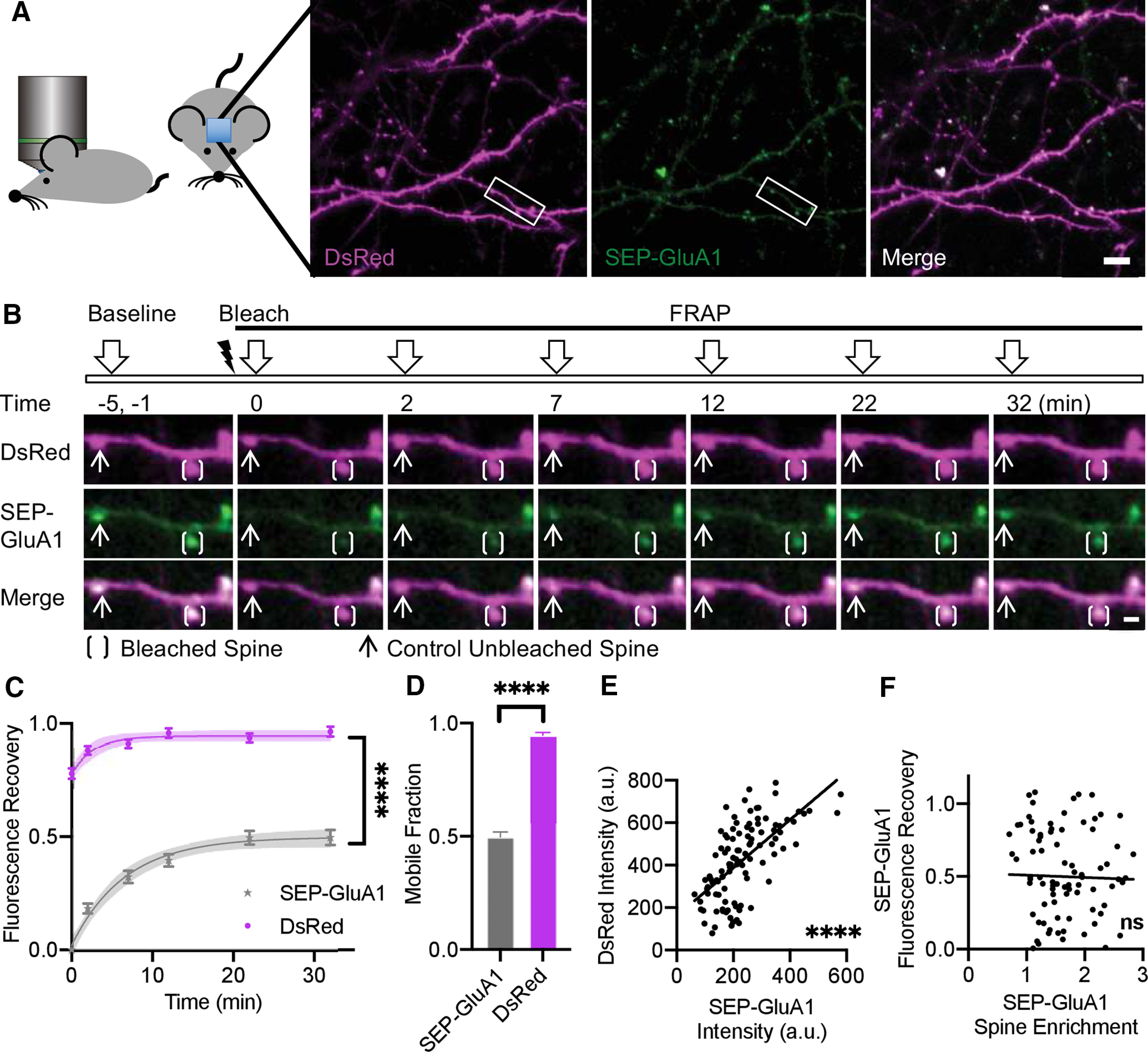
*In vivo* FRAP shows mobile and immobile fractions of GluA1 in cortical neurons. ***A***, Schematic of experimental approach using two-photon imaging of cranial windows in live mice. Representative images of maximum intensity projection (MIP) of 3D z-stack of L5 visual cortex neurons expressing DsRed cell fill (magenta), SEP-GluA1 (green), and myc-GluA2. Scale bar: 10 μm. Area of interest indicated corresponding to panel ***B***. ***B***, Representative MIP image of dendrite with a bleached (bracket) and an unbleached (arrow) spine at indicated time points. Scale bar: 2 μm. ***C***, Fluorescence recovery of SEP-GluA1 versus DsRed cell fill in spines (multifactorial ANOVA; Extended Data Figures 1-3, 1-4). Time points were fitted with an exponential curve indicated by solid line with 95% CI in shaded area. ***D***, Mobile fraction defined by maximum of fluorescence recovery calculated as Ymax of fitted exponential curve displayed in panel ***C*** (*t* test). ***E***, Correlation between initial raw intensity of DsRed cell fill and SEP-GluA1 per spine, Pearson’s correlation *r*^2^ = 0.41. ***F***, Correlation between recovered fraction measured at 32 min postbleach and spine enrichment, Pearson’s correlation *r*^2^ = 0.0006. ***C–F***, *n* = 104 spines, 4 mice, error bars indicate SEM; ****p < 0.0001, ns = not significant. See also Extended Data Figures 1-1, 1-2.

10.1523/ENEURO.0015-21.2021.f1-1 Extended Data Figure 1-1 Fluorescence recovery stable to 42 min postbleach. ***A***, Representative MIP image of dendrite with a bleached (bracket) and an unbleached (arrow) spine at indicated time points. Scale bar: 2 μm. ***B***, Fluorescence recovery of SEP-GluA1 versus DsRed cell fill in spines (multifactorial ANOVA). Time points were fitted with an exponential curve indicated by solid line with 95% CI in shaded areas; *n* = 80 spines, 3 mice. ***C***, Mobile fraction defined by maximum fluorescence recovery calculated as Ymax of fitted exponential curve displayed in panel ***B*** (*t* test). ***D***, Fluorescence recovery without normalization to SEP-GluA1 bleach intensity of SEP-GluA1 versus DsRed cell fill in spines (multifactorial ANOVA); *n* = 104 spines, 4 mice. Error bars indicate SEM, ****p < 0.0001. Download Figure 1-1, EPS file.

### Corticosterone experiments

First, baseline FRAP images were collected as described above. Then at least 1 d after acquisition of baseline images, either corticosterone (water soluble 2-hydroxypropyl-β-cyclodextrin complex, Sigma-Aldrich, dissolved in 0.9% normal saline) at 5 mg/kg or the same volume of 0.9% normal saline was injected intraperitoneally. FRAP images were subsequently collected at 1, 2, and 3 h postinjection in three distinct regions. On a third day, at least 3 d following the previous injection, the injection of the other substance was performed followed by the same imaging protocol. This set of experiments included only mice with neurons electroporated in L2/3 of visual cortex. Only data from animals where all 3 d of data were able to be collected were included in the analysis. Because of the circadian nature of endogenous corticosterone release in mice, all experiments were performed within a 5-h time window between 1 and 6 P.M. to minimize the contribution of innate circadian corticosterone variation to the experimental readout.

### Image analysis

Image processing and analysis were performed in ImageJ following export from Zen (Zeiss). Values measured in ImageJ were subsequently analyzed using custom scripts in MATLAB. All image processing was batched and performed blinded to group or condition, which were later reassigned on performing statistical analyses. Image z-stacks at all time points were maximally projected in the z-dimension. StackRegJ (Jay Unruh, Stowers Institute for Medical Research, Kansas City, MO), a plugin of ImageJ, was used to correct for XY drift. Circular regions of diameter 10 pixels were drawn to define individual bleached spines as well as non-bleached control spines, and integrated values were obtained at all time points. Values were background subtracted. Baseline fluorescence intensity was normalized to 1. FRAP was calculated as the fluorescence increase between time 0 immediately after photobleaching and the indicated time points. Additionally, randomly selected spines in each field that were not bleached were quantified at each time point as controls for bleaching that occurred during image acquisition. The bleached spines were subsequently normalized to the average intensity of the unbleached spines over time.

To measure spine enrichment of SEP-GluA1 we first measured additional ROIs on dendritic regions immediately near each individual spine and offset from areas with hot spots of SEP-GluA1 so as to avoid other synapses. Spine enrichment was defined as the following ratio of background subtracted initial fluorescence intensity: *(SEP_spine_/DsRed_spine_)/(SEP_dendrite_/DsRed_dendrite_)*.

Spine intensity was quantified as DsRed cell fill fluorescence intensity within ROIs in the baseline image for each time series. This was used as a measure of spine size as it has been previously shown to be closely correlated (Holtmaat et al., 2005). Similarly, dendritic intensity was quantified as DsRed cell fill fluorescence intensity within dendritic shaft ROIs and used as a measure of dendritic size. The nearest neighbor distance (NND) was calculated as the shortest distance between all synapses as defined by SEP-GluA1 clusters. These clusters were automatically identified by local fluorescence intensity maxima in the baseline image for each time series so as to avoid bias in identifying synapses. NND was used as a measure of synaptic density whereby shorter NNDs reflects higher density.

### Statistical analysis

Statistical analyses and graphing were performed in Prism 8 (GraphPad software). Where means are shown, errors represent SEM. Box-and-whisker blots represent the median, interquartile range and 5% and 95% of the distribution. Column scatter plots show all individual values with a horizontal line representing the median value. Correlations were fit with a linear regression and assessed for significance using Pearson correlation. Curve fitting of fluorescence recovery was performed using nonlinear regression to fit an exponential association curve defined by Y=YM-(YM-Y0)*exp(-k*x), where YM is the maximum fluorescence, Y0 is starting fluorescence, k is the rate constant of recovery (min^−1^), and x is time in minutes. Outlier removal was performed using the ROUT method with false detection rate Q = 5%. Where curve fits are presented the solid line represents best fit curve and shaded areas represent the 95% confidence interval of the best fit. Pairwise statistical tests were performed using unpaired *t* tests. Comparisons of greater than two conditions were performed using one-way ANOVA. FRAP experiments were compared using multifactorial ANOVA with variables of (1) time across the experiment and (2) experimental conditions. Reported significance on graphs represent whether the interaction of these variables was statistically significant. For statistically significant results, *post hoc* pairwise comparisons were performed using the Sidak’s multiple comparison test. Error for all statistical analyses was set at α = 0.05. Statistical significance was considered at *p* < 0.05. All statistics tables are included in extended data figures.

## Results

We sparsely transfected neurons by *in utero* electroporation of super-ecliptic pHluorin tagged GluA1 (SEP-GluA1), myc-GluA2, and DsRed, which allowed for simultaneous imaging of GluA1-containing AMPARs as well as neuronal morphology. Subsequently, cranial windows were placed for two-photon imaging over the respective cortical regions as previously described and characterized (Zhang et al., 2015; Roth et al., 2020; Tan et al., 2020).

Individual spines were targeted for photobleaching with neighboring spines as unbleached control spines (Fig. 1*A*,*B*). Fluorescence recovery was monitored to 32 min postbleaching for all experiments and remained stable with no significant additional recovery observed up to 42 min postbleaching in a subset of samples (Fig. 1*C*; Extended Data Fig. 1-1*A–C*). Comparison of mobile fractions was subsequently performed using the asymptote (YM) calculated by fit with an exponential association curve (Fig. 1*C*,*D*; Extended Data Figs. 1-3, 1-4). DsRed showed fluorescence recovery to near baseline, reflective of its entirely mobile constituency facilitating exchange. In contrast, SEP-GluA1 recovered to only ∼50% of baseline (Fig. 1*C*,*D*). These findings are most consistent with previous data obtained in dissociated cell culture and suggest that a significant portion of GluA1-containing AMPARs *in vivo* at basal states are contained within an immobile synaptic fraction. They are thus not readily interchanged likely as a result of diffusion restriction via protein interactions and molecular crowding within the postsynaptic architecture.

**Figure 2.**
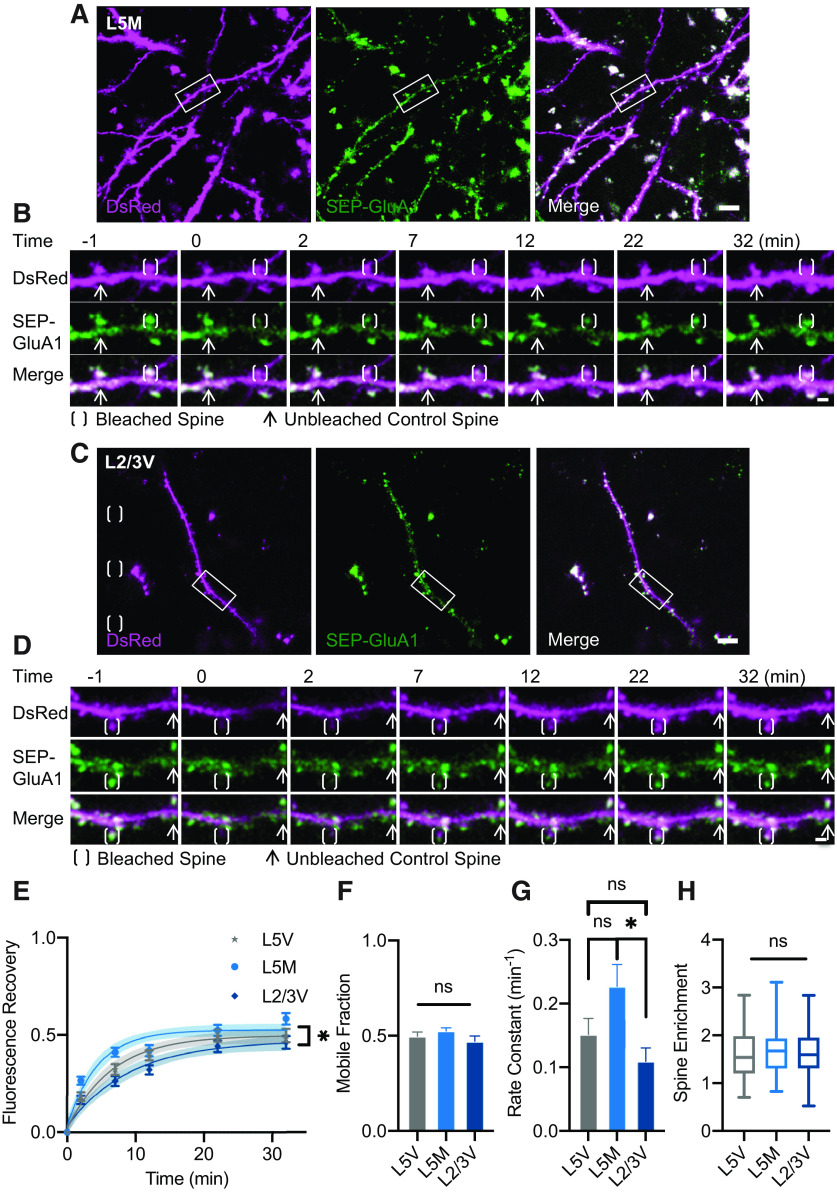
Similar SEP-GluA1 mobility between different cortical regions or layers. ***A***, Representative MIP image of region of L5 motor cortex neurons expressing DsRed cell fill (magenta), SEP-GluA1 (green), and myc-GluA2. Scale bar: 10 μm. Area of interest indicated corresponding to panel ***B***. ***B***, Representative MIP image of bleached spine and fluorescence recovery. Scale bar: 2 μm. ***C***, ***D***, Similar to ***A***, ***B*** in L2/3 of visual cortex neurons (scale bars: 10 and 2 μm, respectively). ***E***, Fluorescence recovery of SEP-GluA1 in spines of L5 visual (L5V), L5 motor (L5M), and L2/3 visual (L2/3V) cortices (multifactorial ANOVA; Extended Data Figures 2-3, 2-4). Time points were fitted with an exponential curve indicated by solid line with 95% CI in shaded area. ***F***, Comparison of SEP-GluA1 mobile fraction across regions/layers defined by maximum fluorescence recovery calculated as Ymax of fitted exponential curves in panel ***E*** (one-way ANOVA, *p* = 0.24; Extended Data Figure 2-5). ***G***, Comparison of SEP-GluA1 recovery rate constant across regions/layers defined by k of fitted exponential curves in panel ***E*** (one-way ANOVA, Sidak’s multiple comparison tests; Extended Data Figure 2-6). ***H***, Comparison of SEP-GluA1 spine enrichment across regions/layers (one-way ANOVA, *p* = 0.40; Extended Data Figure 2-7). ***E–H***, L5V *n* = 104 spines, 4 mice; L5M *n* = 97 spines, 3 mice; L2/3V *n* = 84 spines, 3 mice, *p < 0.05, ns = not significant. See also Extended Data Figures 2-1, 2-2, 2-8, 2-9, 2-10, 2-11, 2-12, 2-13.

10.1523/ENEURO.0015-21.2021.f1-2 Extended Data Figure 1-2 SEP-GluA1 fluorescence recovery not correlated with spine size, shaft size, or synaptic density. ***A***, Correlation between recovered fraction measured at 32 min postbleach and spine intensity, Pearson’s correlation *r*^2^ = 0.04. ***B***, Correlation between recovered fraction measured at 32 min postbleach and dendritic shaft intensity, Pearson’s correlation *r*^2^ = 0.006. ***C***, Correlation between recovered fraction measured at 32 min postbleach and NND, Pearson’s correlation *r*^2^ = 0.02; *n* = 104 spines, 4 mice, ns = not significant. Download Figure 1-2, EPS file.

**Figure 3:**
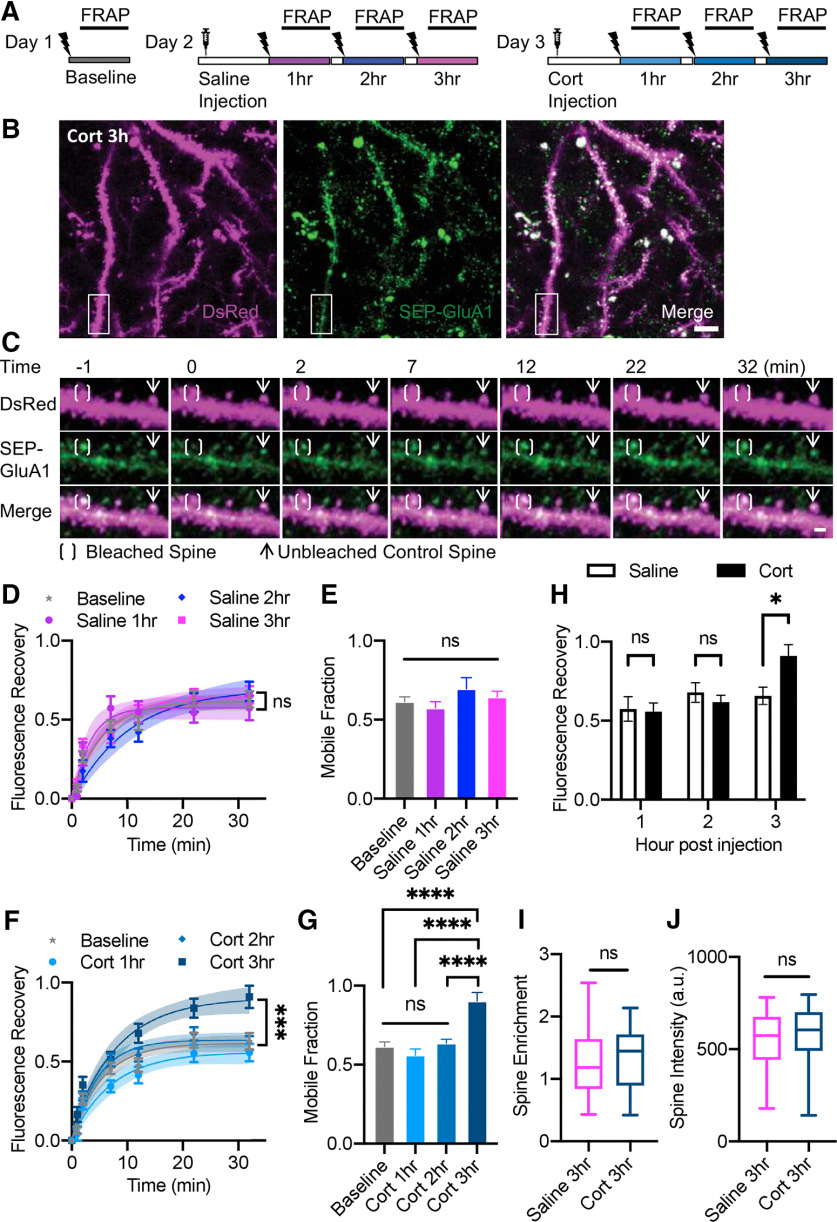
Corticosterone increases GluA1 mobility within spines. ***A***, Schematic of experimental design: FRAP performed on same cohort of mice across three separate days to obtain baseline and measurements at 1, 2, and 3 h postinjection of saline and corticosterone. ***B***, Representative MIP image of L2/3 visual cortex neurons expressing DsRed cell fill (magenta), SEP-GluA1 (green), and myc-GluA2 3 h postinjection of saline. Scale bar: 10 μm. Area of interest indicated corresponding to panel ***C***. ***C***, Representative MIP image of bleached (bracket) and unbleached (arrow) spines and fluorescence recovery. Scale bar: 2 μm. See also Extended Data Figure 3-1. ***D***, Fluorescence recovery of SEP-GluA1 in spines at baseline (*n* = 106 spines) and at 1 h (*n* = 45 spines), 2 h (*n* = 51 spines), and 3 h (*n* = 53 spines) postinjection of saline (multifactorial ANOVA; Extended Data Figures 3-3, 3-4). Time points were fitted with an exponential curve indicated by solid line with 95% CI in shaded area. ***E***, Comparison of mobile fraction at baseline and indicated times postinjection of saline defined by maximum fluorescence recovery calculated as Ymax of fitted exponential curves displayed in panel ***D*** (one-way ANOVA; Extended Data Figure 3-5). ***F***, ***G***, Similar to ***D***, ***E*** at baseline and 1 h (*n* = 43 spines), 2 h (*n* = 55 spines), and 3 h (*n* = 51 spines) postinjection of corticosterone [Cort; multifactorial ANOVA for ***F*** (Extended Data Figure 3-6), one-way ANOVA with Sidak’s multiple comparison tests for ***G*** (Extended Data Figure 3-7)]. ***H***, Comparison of fluorescence recovery at times postinjection of corticosterone versus saline (two-way ANOVA with Sidak’s multiple comparison test; Extended Data Figure 3-8). ***I***, Comparison of spine enrichment at 3 h postinjection (*t* test, *p* =0.43). ***J***, Comparison of spine DsRed intensity normalized to nearby dendritic shaft DsRed intensity at 3 h postinjection (*t* test, *p* = 0.98). ***D–J***, *n* = 5 mice, error bars indicate SEM, ****p < 0.0001, ***p < 0.001, *p < 0.05, ns = not significant. See also Extended Data Figure 3-2.

10.1523/ENEURO.0015-21.2021.f1-3 Extended Data Figure 1-3 Exponential plateau curve fit for fluorescence recovery (Fig. 1c). Download Figure 1-3, DOCX file.

10.1523/ENEURO.0015-21.2021.f1-4 Extended Data Figure 1-4 Multifactorial ANOVA corresponding to comparison of fluorescence recovery between SEP-GluA1 and DsRed cell fill (Fig. 1c). Download Figure 1-4, DOCX file.

Consistent with previous data (Zhang et al., 2015), the initial fluorescence intensity of DsRed, reflective of spine size, strongly correlated with initial intensity of SEP-GluA1, reflective of spine AMPAR content (Fig. 1*E*). Interestingly, the relative concentration of SEP-GluA1 in spines relative to dendrites, defined as SEP-GluA1 spine enrichment, did not correlate with the magnitude of SEP-GluA1 fluorescence recovery (Fig. 1*F*), suggesting that the mobile fraction of GluA1-containing AMPARs is independent of the spine GluA1 level, also consistent with previous findings (Li et al., 2016). We found that the mobile fraction was also not correlated with spine DsRed intensity, an estimate of spine size; dendritic shaft DsRed intensity, an estimate of dendritic size; or NND of synapses, an estimate of synapse density (Extended Data Fig. 1-2).

Additionally, we sought to assess whether any differences existed between cortical areas responsible for sensory input, specifically the visual cortex, compared with motor output in the motor cortex. Thus, we compared AMPAR dynamics between neurons in L5 of motor and visual cortices (Fig. 2*A*,*B*). Furthermore, as different lamina within the cortical columnar stack possess unique inputs and feedback connections as well as distinct morphologic and electrophysiological properties (Thomson and Lamy, 2007; Gouwens et al., 2019), we also investigated whether AMPAR mobility differed within different cortical layers through comparison of neurons in L5 versus L2/3 of visual cortex (Fig. 2*C*,*D*). Overall, the total mobile fraction and spine enrichment of SEP-GluA1 in different regions and layers were comparable (Fig. 2*E*,*F*,*H*; Extended Data Figs. 2-3, 2-4, 2-5, 2-7). However, we did observe a significant difference in the time courses of SEP-GluA1 FRAP curves with a faster rate constant of recovery within L5 of motor cortex compared with L2/3 of visual cortex (Fig. 2*E*,*G*; Extended Data Fig. 2-6). In parallel, we found that spine DsRed intensity, an estimate of spine size, varied across the regions and layers in a similar pattern as recovery rate (Extended Data Figs. 2-1*A*, 2-8). To further assess this relationship, we pooled data from all spines across regions and layers and divided the spines into three equal bins to compare small, medium, and large spines. Indeed, we found that spine size was inversely related to recovery rate such that larger spines recovered faster than smaller spines (Extended Data Figs. 2-1*D*,*E*, 2-11, 2-12, 2-13). Meanwhile, measures of dendritic size and synaptic density did show notable differences between the different regions and layers, consistent with some prior observations (Konur et al., 2003; Holtmaat et al., 2005), but these differences did not parallel those observed in recovery rate (Extended Data Figs. 2-1*B*,*C*, 2-9, 2-10). No differences were seen between groups for FRAP of DsRed cell fill (Extended Data Fig. 2-2).

10.1523/ENEURO.0015-21.2021.f2-1 Extended Data Figure 2-1 SEP-GluA1 recovery rate inversely related to spine size. ***A***, Comparison of spine intensity between spines of L5 visual (L5V), L5 motor (L5M), and L2/3 visual (L2/3V) cortices (one-way ANOVA; Extended Data Fig. 2-8). ***B***, Similar as panel ***A*** for shaft intensity (Extended Data Fig. 2-9). ***C***, Similar as panel ***A*** for synapse NND (Extended Data Fig. 2-10). ***D***, Fluorescence recovery of SEP-GluA1 in pooled spines separated into equal bins by spine intensity (multifactorial ANOVA; Extended Data Figs. 2-1, 2-11). Time points were fitted with an exponential curve indicated by solid line with 95% CI in shaded areas. ***E***, Comparison between spine sizes of recovery rate constant defined by k of fitted exponential curves in panel ***D*** (one-way ANOVA, Sidak’s multiple comparison tests; Extended Data Fig. 2-13). L5V *n* = 104 spines, 4 mice; L5M *n* = 97 spines, 3 mice; L2/3V *n* = 84 spines, 3 mice. Error bars indicate SEM, **p < 0.01, ***p < 0.001, ****p < 0.0001, ns = not significant. Download Figure 2-1, EPS file.

10.1523/ENEURO.0015-21.2021.f2-2 Extended Data Figure 2-2 Fluorescence recovery of DsRed similar in different cortical regions and layers. Fluorescence recovery of DsRed in spines of L5 visual (L5V), L5 motor (L5M), and L2/3 visual (L2/3V) cortices (multifactorial ANOVA, *p* = 0.66). Time points were fitted with an exponential curve indicated by solid line with 95% CI in shaded areas. L5V *n* = 104 spines, 4 mice; L5M *n* = 97 spines, 3 mice; L2/3V *n* = 84 spines, 3 mice, ns = not significant. Download Figure 2-2, EPS file.

10.1523/ENEURO.0015-21.2021.f2-3 Extended Data Figure 2-3 Exponential curve fit for fluorescence recovery across regions/layer (Fig. 2e). Download Figure 2-3, DOCX file.

10.1523/ENEURO.0015-21.2021.f2-4 Extended Data Figure 2-4 Multifactorial ANOVA corresponding to comparison of fluorescence recovery across regions/layer (Fig. 2e). Download Figure 2-4, DOCX file.

10.1523/ENEURO.0015-21.2021.f2-5 Extended Data Figure 2-5 1-way ANOVA corresponding to comparison of mobile fraction across regions/layers (Fig. 2f). Download Figure 2-5, DOCX file.

10.1523/ENEURO.0015-21.2021.f2-6 Extended Data Figure 2-6 1-way ANOVA corresponding to comparison of recovery rate constant across regions/layers with Sidak's multiple comparisons test (Fig. 2g). Download Figure 2-6, DOCX file.

10.1523/ENEURO.0015-21.2021.f2-7 Extended Data Figure 2-7 1-way ANOVA corresponding to comparison of spine enrichment across regions/layers (Fig. 2h). Download Figure 2-7, DOCX file.

10.1523/ENEURO.0015-21.2021.f2-8 Extended Data Figure 2-8 1-way ANOVA corresponding to comparison of spine intensity across regions/layers with Sidak's multiple comparisons test (Fig. 2-1a). Download Figure 2-8, DOCX file.

10.1523/ENEURO.0015-21.2021.f2-9 Extended Data Figure 2-9 1-way ANOVA corresponding to comparison of shaft intensity across regions/layers with Sidak's multiple comparisons test (Fig. 2-1b). Download Figure 2-9, DOCX file.

10.1523/ENEURO.0015-21.2021.f2-10 Extended Data Figure 2-10 1-way ANOVA corresponding to comparison of synapse nearest neighbor distance across regions/layers with Sidak's multiple comparisons test (Fig. 2-1c). Download Figure 2-10, DOCX file.

10.1523/ENEURO.0015-21.2021.f2-11 Extended Data Figure 2-11 Exponential curve fit for fluorescence recovery across spine sizes (Fig. 2-1d). Download Figure 2-11, DOCX file.

10.1523/ENEURO.0015-21.2021.f2-12 Extended Data Figure 2-12 Multifactorial ANOVA corresponding to comparison of fluorescence recovery across spine sizes (Fig. 2-1d). Download Figure 2-12, DOCX file.

10.1523/ENEURO.0015-21.2021.f2-13 Extended Data Figure 2-13 1-way ANOVA corresponding to comparison of FRAP recovery rate across spine sizes with Sidak's multiple comparisons test (Fig. 2-1e). Download Figure 2-13, DOCX file.

Finally, the mobility and trafficking of GluA1 within synapses have been shown to be regulated by various mechanisms, including by glucocorticoids. Specifically, in rodents, corticosterone, the main glucocorticoid, has been found to have effects on synaptic transmission and plasticity, which may underlie the influence of stress on learning and memory (Krugers et al., 2010; Timmermans et al., 2013; McEwen et al., 2015). Past studies have found that intraperitoneal injection of corticosterone increases spine formation and elimination in cortical neurons within hours of injection (Liston and Gan, 2011). Moreover, timing of corticosterone injections relative to innate circadian corticosterone peaks and troughs affects learning (Liston et al., 2013). Furthermore, application of corticosterone to cultured hippocampal neurons increased both GluA1-containing and GluA2-containing AMPAR surface mobility and surface expression (Heine et al., 2008; Martin et al., 2009). Particularly pronounced effects on AMPAR mobility were seen 3 h following corticosterone application whereby FRAP imaging revealed complete FRAP, suggesting complete loss of the immobile pool of synaptic AMPARs following treatment (Martin et al., 2009).

We therefore sought to determine whether corticosterone would have similar effects on AMPAR mobility in synapses *in vivo*. We performed repeated measures comparisons of AMPAR mobility within different subsets of dendritic spines of the same mice intraperitoneally injected with corticosterone (dissolved in 0.9% normal saline) versus normal saline on different days up to 3 h postinjection compared with baseline with no injection (Fig. 3; Extended Data Fig. 3-1). We found that SEP-GluA1 fluorescence recovery significantly increased to ∼90% 3 h following injection of corticosterone compared with similar fluorescence recovery levels between baseline and 1 to 2 h following injection (Fig. 3*F*,*G*; Extended Data Figs. 3-6, 3-7). In contrast, injection of saline produced no such change (Fig. 3*D*,*E*; Extended Data Figs. 3-3, 3-4, 3-5). Comparison of fluorescence recovery at 32 min after photobleaching showed significantly higher recovery of SEP-GluA1 3 h following corticosterone injection compared with 3 h following saline injection (Fig. 3*H*; Extended Data Fig. 3-8). This suggests that corticosterone injection causes a shift from a near equal split between mobile and immobile pools of GluA1-containing receptors to an almost entirely mobile pool of GluA1-containing receptors within cortical synapses, which is consistent with that previously seen within dissociated hippocampal cultures (Groc et al., 2008; Martin et al., 2009). This observed difference in receptor mobility was not associated with differences between SEP-GluA1 spine enrichment or spine DsRed intensity, suggesting corticosterone induced increase in mobile fraction was unlikely because of change in receptor spine enrichment or spine size (Fig. 3*I*,*J*). No differences were observed in FRAP of DsRed cell fill FRAP (Extended Data Fig. 3-2).

10.1523/ENEURO.0015-21.2021.f3-1 Extended Data Figure 3-1 Fluorescence recovery of corticosterone versus saline injection. ***A***, Representative MIP images of region of L2/3 visual cortex neurons expressing DsRed cell fill (magenta), SEP-GluA1 (green), and myc-GluA2 at baseline. Scale bar: 10 μm. Area of interest indicated corresponding to panel ***B***. ***B***, Representative MIP image of bleached (bracket) and unbleached (arrow) spines at baseline FRAP. Scale bar: 2 μm. ***C***, ***D***, same as ***A***, ***B*** for 3 h postinjection of saline. Download Figure 3-1, EPS file.

10.1523/ENEURO.0015-21.2021.f3-2 Extended Data Figure 3-2 Fluorescence recovery of corticosterone versus saline injection. ***A***, Fluorescence recovery of DsRed in spines of L2/3 visual (multifactorial ANOVA, *p* = 0.45) at baseline (*n* = 106 spines) and at 1 h (*n* = 43 spines), 2 h (*n* = 55 spines), and 3 h (*n* = 51 spines) postinjection of corticosterone. Time points were fitted with an exponential curve indicated by solid line with 95% CI in shaded area. ***B***, Similar as a for at 1 h (*n* = 45 spines), 2 h (*n* = 51 spines), and 3 h (*n* = 53 spines) postinjection of saline. Download Figure 3-2, EPS file.

10.1523/ENEURO.0015-21.2021.f3-3 Extended Data Figure 3-3 Exponential curve fit for fluorescence recovery in baseline, corticosterone-, or saline- treated mice (Fig. 3d, 3f). Download Figure 3-3, DOCX file.

10.1523/ENEURO.0015-21.2021.f3-4 Extended Data Figure 3-4 Multifactorial ANOVA corresponding to comparison of fluorescence recovery between baseline and times after saline injection and Sidak's multiple comparisons (Fig. 3d). Download Figure 3-4, DOCX file.

10.1523/ENEURO.0015-21.2021.f3-5 Extended Data Figure 3-5 1-way ANOVA corresponding to comparison of mobile fraction baseline and times after saline injection (Fig. 3e). Download Figure 3-5, DOCX file.

10.1523/ENEURO.0015-21.2021.f3-6 Extended Data Figure 3-6 Multifactorial ANOVA corresponding to comparison of fluorescence recovery between baseline and times after corticosterone injection and Sidak's multiple comparisons (Fig. 3f). Download Figure 3-6, DOCX file.

10.1523/ENEURO.0015-21.2021.f3-7 Extended Data Figure 3-7 1-way ANOVA and Sidak's multiple comparisons test corresponding to comparison of mobile fraction baseline and times after corticosterone injection (Fig. 3g). Download Figure 3-7, DOCX file.

10.1523/ENEURO.0015-21.2021.f3-8 Extended Data Figure 3-8 2-way ANOVA and Sidak's multiple comparison test corresponding to comparison between fluorescence recovery at 32 min for corticosterone vs saline injection at 1-, 2-, and 3-hours post-injection (Fig. 3h). Download Figure 3-8, DOCX file.

## Discussion

This study shows that within cortical synapses *in vivo*, there exist populations of mobile and immobile GluA1-containing receptors in basal states. This is generally consistent with previous findings in dissociated cell culture studies (Ashby et al., 2006; Bats et al., 2007; Heine et al., 2008; Frischknecht et al., 2009; Martin et al., 2009; Arendt et al., 2010). On the other hand, this is incongruent with findings in organotypic slice cultures that suggest GluA1-containing receptors are entirely mobile in basal states and are only incorporated into immobile synaptic fractions during LTP (Hayashi et al., 2000; Kopec et al., 2006; Makino and Malinow, 2009). Additionally, the existence of an immobile fraction of GluA1-containing receptors suggests that these receptors are present within synapses and do not solely reside within spine surface receptors pools. This would also suggest that the presence of the SEP tag on recombinant GluA1-containing receptors does not intrinsically interfere with its trafficking to synapses as has been suggested (Díaz-Alonso et al., 2017). These differences likely result from the different preparations and experimental conditions, highlighting the need for experimentation within more intact systems.

We further observe that the proportion of GluA1-containing receptors is similar across different cortical areas of the brain as well as different layers within the visual cortex. Our data suggest that across the different regions and layers, larger spine sizes may be associated with higher rates of receptor mobility. This finding is consistent with previous work showing that spine morphology affects diffusion of molecules within spines, and more specifically, that spine volume positively correlates with FRAP recovery rate (Tønnesen et al., 2014). As such, spine morphology is proposed to confer compartmentalized signaling capacity to synapses allowing synaptic strength to be regulated independently from neighboring synapses. Moreover, we find that the proportion of mobile to immobile GluA1-containing receptors may be altered by corticosterone in a time-dependent manner. This suggests that AMPAR mobility is a fluid parameter within cortical neurons that may be regulated by stress to modulate basal synaptic strength or synaptic plasticity. Overall, these findings point to common basic properties of glutamatergic receptors that likely reflect their tight regulation within synapses given their critical function within the nervous system.

## Synthesis

Reviewing Editor: Lindsay De Biase, University of California, Los Angeles Decisions are customarily a result of the Reviewing Editor and the peer reviewers coming together and discussing their recommendations until a consensus is reached. When revisions are invited, a fact-based synthesis statement explaining their decision and outlining what is needed to prepare a revision will be listed below. The following reviewer(s) agreed to reveal their identity: NONE.

Both reviewers were in strong agreement that this work provides valuable in vivo confirmation of features of AMPA receptor mobility that have so far only been demonstrated in vitro. Reviewer 1 summarizes “AMPA receptor mobility, particularly of the GluA1 subunit, is thought to underlie many aspects of synapse plasticity. FRAP is the dominant method of analyzing this mobility, but previous work using FRAP in vitro in brain slices and neuron cultures has shown several contradictions and inconsistencies as pointed out by the authors, some of which may arise from the preparation. Receptor mobility has not been directly measured in vivo previously. Thus, this paper fills an important gap in the literature.”

There were several relatively minor concerns that reviewers raised:

1. There is no mention of blinding in the experiment acquisition or analysis, but presumably selecting spines for bleaching or the non-bleached controls spines for analysis each carry substantial risk of bias. A statement describing the blinding strategy should be included. It would be disappointing if this was not, but if it weren't, it could be addressed with new experiments or with some post hoc re-analysis testing whether the choice of spines influences the results.

2. The difference in recovery rate in Fig 2G is left entirely out of the discussion, but holds some interest. Are there explanations or interpretations the authors could propose? The morphology of the exemplar dendrites in Fig 2A and C seem different from one another, particularly compared to Fig 1B, and one wonders whether the neuron or dendrite choice could explain the differences. Did the FRAP recover rate or mobile fraction depend on the neighboring spine density or the dendrite shaft diameter?

3. Conducting such experiments in vivo is admirable. However, the design still utilizes an overexpression system, and some discussion should be given to the potential artifacts that this might risk incurring. The use of overexpression should be mentioned in the abstract to avoid confusion given the increasing availability of knock-ins and gene editing.

4. The statement on line 14 is a bit too bald: “With this system we are able to demonstrate that there are both mobile and immobile AMPAR fractions.” The authors undoubtedly don't mean to imply that these subpopulations had not been observed previously, but they should avoid the implication.

5. In Fig 1C, the main data points showing the means and the error bars on these plots are very small. In the reviewer version of Fig 3d and 3f, the confidence interval was not reproduced properly.

## Author Response

Dear Dr. De Biase,

Thank you for your consideration for publication of our manuscript in eNeuro and for the

thoughtful comments from the reviewers. We have addressed each point individually below and

hope it is to your satisfaction.

In response to the reviewers individual points:

1. There is no mention of blinding in the experiment acquisition or analysis, but

presumably selecting spines for bleaching or the non-bleached controls spines for

analysis each carry substantial risk of bias. A statement describing the blinding strategy

should be included. It would be disappointing if this was not, but if it weren't, it could be

addressed with new experiments or with some post hoc re-analysis testing whether the

choice of spines influences the results.

Now included in the methods section:

All image processing was batched and performed blind to group or condition, which were later

reassigned on performing statistical analyses.

Additionally, to test whether a different selection of non-bleached control spines for analysis

affects the results, for the L5V FRAP images, we picked out an additional set of unbleached

spines to double the total number of unbleached spines selected for each image. From this total

pool, we then used a random permutation generator to select half of the spines to use as the

control spines. We then repeated the fitting, and found no significant difference between using

the original set of control spines and the new random selection of control spines (fit shown

below).

2. The difference in recovery rate in Fig 2G is left entirely out of the discussion, but holds

some interest. Are there explanations or interpretations the authors could propose? The

morphology of the exemplar dendrites in Fig 2A and C seem different from one another,

particularly compared to Fig 1B, and one wonders whether the neuron or dendrite choice 2

could explain the differences. Did the FRAP recover rate or mobile fraction depend on

the neighboring spine density or the dendrite shaft diameter?

This is a very interesting question posed by the reviewers and based on their suggestions, we

went back to compare spine and dendritic characteristics with regards to mobile fraction and

recovery rate. We measured spine size using baseline DsRed cell fill fluorescent intensity within

spines; dendritic thickness using DsRed cell fill fluorescent intensity within dendritic regions

close to bleached spines; and spine density using nearest neighbor distance (NND) for each

bleached spine so shorter NNDs should reflect higher density.

Within the dataset of L5V spines, we looked at the correlation between each of these properties

with fluorescence recovery and found no significant correlations. This is consistent with previous

studies showing similar mobile fractions across different PSD areas (Li et al. 2016) as spine size

and PSD size are tightly correlated.

This data is now described in the text and included in the supplemental figures.

We did not look at similar correlations between spine and dendritic properties with recovery rate

constants because fitting an exponential recovery curve needed to calculate rate constants to

the limited data points of each individual spine data is not reliably accurate. Therefore, we

address the rate constant question below with pooled data both between and across

region/layer groups.

In comparing these spine and dendritic characteristics between the different regions and layers,

there were notable differences that may reflect intrinsic differences in these properties between

the different regions and layers, though may also reflect some degree of sampling artifact. For

example, Konur et al (2003) and Holtmaat et al (2005) also have observed smaller interspine

distances or higher spine density in cortical layer 2/3 neurons than layer 5 neurons, though

there is high inter-cell variability.

Of these properties, the only one that was notable for a significant difference between L5M and

L2/3V as seen in rate constants is spine size. Indeed, previous work has suggested a positive

relationship between spine volume and FRAP recovery rate (Tonnesen et al 2014). To further

assess whether this correlation holds, we pooled all of the FRAP data across regions and layers

(N=285 spines) and divided the spines into 3 equal bins (N=95 spines each) noted as small,

medium, and large. Then, fitting these groups with an exponential recovery function showed that

larger spines recovered faster than smaller spines. This data is now described in the text and

included in the supplemental figures.

To be more consistent in measures of spine size shown, Figure 3j was changed from

normalized spine intensity to raw spine intensity. This did not change the conclusions, and the

comparison is shown below.

3. Conducting such experiments in vivo is admirable. However, the design still utilizes an

overexpression system, and some discussion should be given to the potential artifacts

that this might risk incurring. The use of overexpression should be mentioned in the

abstract to avoid confusion given the increasing availability of knock-ins and gene

editing.

"Here we use two-photon-FRAP to study AMPAR mobility within individual dendritic spines in

live animals using an overexpression vector.”

4. The statement on line 14 is a bit too bald: “With this system we are able to

demonstrate that there are both mobile and immobile AMPAR fractions.” The authors

undoubtedly don't mean to imply that these subpopulations had not been observed

previously, but they should avoid the implication.

"Here within this system we are able to confirm that there are both mobile and immobile AMPAR

fractions in vivo and that these fractions are similar across different cortical regions and layers.”

5. In Fig 1C, the main data points showing the means and the error bars on these plots

are very small. In the reviewer version of Fig 3d and 3f, the confidence interval was not

reproduced properly.

Thank you for pointing out these problems. We hope these are sufficiently visible on the final

figures submitted.

Finally, in going back through the data to address the reviewer questions, we noticed that there

was unfortunately an error in one of the images that was included in the L5V group - an image

that was excluded due to a missing time point (image n=14 spines) was misfiled and

accidentally switched with an image that was supposed to be included (image n=13 spines). We

subsequently repeated all of the analyses that this error affected both including the correct

image (total n = 104 spines) and without the correct image (total n = 91 spines), the results of

which were quite similar. It did not change any of the conclusions from the original draft, but it

did change the graphs, which we have included together below for easy comparison and full

transparency. Of note, it did change the statistical significance in figure 2g in which the rate

constant difference between L5V and L2/3V is no longer statistically significant, but the

difference between L5M and L2/3V remains statistically significant. We apologize for not having

noticed this error earlier, and if there are any other concerns related to it, we are more than

happy to address them.

Thanks again for your consideration.
