# Supplementary material for: AMPA Receptors Exist in Tunable Mobile and Immobile Synaptic Fractions In Vivo
Source: eNeuro. 2021 May 14;8(3):ENEURO.0015-21.2021. doi: 10.1523/ENEURO.0015-21.2021 (PMC8143022; doi:10.1523/ENEURO.0015-21.2021)
Supplement: Extended Data Figure 1-3 — Exponential plateau curve fit for fluorescence recovery (Fig. 1c). Download Figure 1-3, DOCX file. [file enu-eN-REV-0015-21-s04.docx]

Figure 1-3 | Exponential plateau curve fit for fluorescence recovery (Fig. 1c)

|  | SEP-GluA1 | DsRed |
| --- | --- | --- |
| Best-fit values |  |  |
| YM | 0.4973 | 0.9474 |
| Y0 | 0.01990 | 0.7826 |
| k | 0.1514 | 0.3664 |
| 95% CI (profile likelihood) |  |  |
| YM | 0.4550 to 0.5484 | 0.9241 to 0.9768 |
| Y0 | -0.02410 to 0.06292 | 0.7406 to 0.8239 |
| k | 0.1052 to 0.2174 | 0.1392 to 0.9847 |
| Goodness of Fit |  |  |
| Degrees of Freedom | 558 | 591 |
| R squared | 0.3512 | 0.08182 |
| Sum of Squares | 33.36 | 24.96 |
| Sy.x | 0.2445 | 0.2055 |
