# Supplementary material for: AMPA Receptors Exist in Tunable Mobile and Immobile Synaptic Fractions In Vivo
Source: eNeuro. 2021 May 14;8(3):ENEURO.0015-21.2021. doi: 10.1523/ENEURO.0015-21.2021 (PMC8143022; doi:10.1523/ENEURO.0015-21.2021)
Supplement: Extended Data Figure 1-4 — Multifactorial ANOVA corresponding to comparison of fluorescence recovery between SEP-GluA1 and DsRed cell fill (Fig. 1c). Download Figure 1-4, DOCX file. [file enu-eN-REV-0015-21-s05.docx]

Figure 1-4 | Multifactorial ANOVA corresponding to comparison of fluorescence recovery between SEP-GluA1 and DsRed cell fill (Fig. 1c)

| Fixed effects (type III) | P value | F (DFn, DFd) |
| --- | --- | --- |
| Time | <0.0001 | F (3.962, 744.1) = 97.70 |
| Construct | <0.0001 | F (1, 204) = 758.4 |
| Time x Construct | <0.0001 | F (5, 939) = 23.73 |
