# Supplementary material for: AMPA Receptors Exist in Tunable Mobile and Immobile Synaptic Fractions In Vivo
Source: eNeuro. 2021 May 14;8(3):ENEURO.0015-21.2021. doi: 10.1523/ENEURO.0015-21.2021 (PMC8143022; doi:10.1523/ENEURO.0015-21.2021)
Supplement: Extended Data Figure 2-3 — Exponential curve fit for fluorescence recovery across regions/layer (Fig. 2e). Download Figure 2-3, DOCX file. [file enu-eN-REV-0015-21-s08.docx]

Figure 2-3 | Exponential curve fit for fluorescence recovery across regions/layer (Fig. 2e)

|  | L5V | L5M | L2/3V |
| --- | --- | --- | --- |
| Best-fit values |  |  |  |
| YM | 0.4973 | 0.5247 | 0.4707 |
| Y0 | 0.01990 | 0.02823 | 0.02907 |
| k | 0.1514 | 0.2268 | 0.1094 |
| 95% CI (profile likelihood) |  |  |  |
| YM | 0.4550 to 0.5484 | 0.4858 to 0.5714 | 0.4210 to 0.5436 |
| Y0 | -0.02410 to 0.06292 | -0.02349 to 0.07777 | -0.01127 to 0.06850 |
| k | 0.1052 to 0.2174 | 0.1448 to 0.3671 | 0.07004 to 0.1624 |
| Goodness of Fit |  |  |  |
| Degrees of Freedom | 558 | 555 | 452 |
| R squared | 0.3512 | 0.3865 | 0.3743 |
| Sum of Squares | 33.36 | 31.13 | 19.47 |
| Sy.x | 0.2445 | 0.2368 | 0.2075 |
