# Supplementary material for: AMPA Receptors Exist in Tunable Mobile and Immobile Synaptic Fractions In Vivo
Source: eNeuro. 2021 May 14;8(3):ENEURO.0015-21.2021. doi: 10.1523/ENEURO.0015-21.2021 (PMC8143022; doi:10.1523/ENEURO.0015-21.2021)
Supplement: Extended Data Figure 2-4 — Multifactorial ANOVA corresponding to comparison of fluorescence recovery across regions/layer (Fig. 2e). Download Figure 2-4, DOCX file. [file enu-eN-REV-0015-21-s09.docx]

Figure 2-4 | Multifactorial ANOVA corresponding to comparison of fluorescence recovery across regions/layer (Fig. 2e)

| Fixed effects (type III) | P value | P value summary | F (DFn, DFd) |
| --- | --- | --- | --- |
| Time | <0.0001 | **** | F (4.050, 1032) = 288.9 |
| Regions/layers | 0.0011 | ** | F (2, 282) = 6.960 |
| Time x Regions/layers | 0.0472 | * | F (10, 1274) = 1.857 |
|  |  |  |  |
| Random effects | SD | Variance |  |
| Subject | 0.1391 | 0.01935 |  |
| Residual | 0.1843 | 0.03398 |  |
