# Supplementary material for: AMPA Receptors Exist in Tunable Mobile and Immobile Synaptic Fractions In Vivo
Source: eNeuro. 2021 May 14;8(3):ENEURO.0015-21.2021. doi: 10.1523/ENEURO.0015-21.2021 (PMC8143022; doi:10.1523/ENEURO.0015-21.2021)
Supplement: Extended Data Figure 2-5 — 1-way ANOVA corresponding to comparison of mobile fraction across regions/layers (Fig. 2f). Download Figure 2-5, DOCX file. [file enu-eN-REV-0015-21-s10.docx]

Figure 2-5 | 1-way ANOVA corresponding to comparison of mobile fraction across regions/layers (Fig. 2f)

| ANOVA table | SS | DF | MS | F (DFn, DFd) | P value |
| --- | --- | --- | --- | --- | --- |
| Treatment (between columns) | 0.7300 | 2 | 0.3650 | F (2, 1565) = 1.440 | P=0.2372 |
| Residual (within columns) | 396.7 | 1565 | 0.2535 |  |  |
| Total | 397.4 | 1567 |  |  |  |
