# Supplementary material for: AMPA Receptors Exist in Tunable Mobile and Immobile Synaptic Fractions In Vivo
Source: eNeuro. 2021 May 14;8(3):ENEURO.0015-21.2021. doi: 10.1523/ENEURO.0015-21.2021 (PMC8143022; doi:10.1523/ENEURO.0015-21.2021)
Supplement: Extended Data Figure 2-6 — 1-way ANOVA corresponding to comparison of recovery rate constant across regions/layers with Sidak's multiple comparisons test (Fig. 2g). Download Figure 2-6, DOCX file. [file enu-eN-REV-0015-21-s12.docx]

Figure 2-6 | 1-way ANOVA corresponding to comparison of recovery rate constant across regions/layers with Sidak’s multiple comparisons test (Fig. 2g)

| ANOVA table | SS | DF | MS | F (DFn, DFd) | P value |
| --- | --- | --- | --- | --- | --- |
| Treatment (between columns) | 3.625 | 2 | 1.813 | F (2, 1565) = 4.369 | P=0.0128 |
| Residual (within columns) | 649.3 | 1565 | 0.4149 |  |  |
| Total | 652.9 | 1567 |  |  |  |

| Sidak's multiple comparisons test | Mean Diff. | 95.00% CI of diff. | Summary | Adjusted P Value |
| --- | --- | --- | --- | --- |
| L5V vs. L5M | -0.07540 | -0.1676 to 0.01682 | ns | 0.1449 |
| L5V vs. L2/3V | 0.04200 | -0.05533 to 0.1393 | ns | 0.6607 |
| L5M vs. L2/3V | 0.1174 | 0.01994 to 0.2148 | * | 0.0121 |
