# Supplementary material for: AMPA Receptors Exist in Tunable Mobile and Immobile Synaptic Fractions In Vivo
Source: eNeuro. 2021 May 14;8(3):ENEURO.0015-21.2021. doi: 10.1523/ENEURO.0015-21.2021 (PMC8143022; doi:10.1523/ENEURO.0015-21.2021)
Supplement: Extended Data Figure 2-7 — 1-way ANOVA corresponding to comparison of spine enrichment across regions/layers (Fig. 2h). Download Figure 2-7, DOCX file. [file enu-eN-REV-0015-21-s11.docx]

Figure 2-7 | 1-way ANOVA corresponding to comparison of spine enrichment across regions/layers (Fig. 2h)

| ANOVA table | SS | DF | MS | F (DFn, DFd) | P value |
| --- | --- | --- | --- | --- | --- |
| Treatment (between columns) | 0.2422 | 2 | 0.1211 | F (2, 276) = 0.5005 | P=0.6068 |
| Residual (within columns) | 66.78 | 276 | 0.2420 |  |  |
| Total | 67.03 | 278 |  |  |  |
