# Supplementary material for: AMPA Receptors Exist in Tunable Mobile and Immobile Synaptic Fractions In Vivo
Source: eNeuro. 2021 May 14;8(3):ENEURO.0015-21.2021. doi: 10.1523/ENEURO.0015-21.2021 (PMC8143022; doi:10.1523/ENEURO.0015-21.2021)
Supplement: Extended Data Figure 2-8 — 1-way ANOVA corresponding to comparison of spine intensity across regions/layers with Sidak's multiple comparisons test (Fig. 2-1a). Download Figure 2-8, DOCX file. [file enu-eN-REV-0015-21-s13.docx]

Figure 2-8 | 1-way ANOVA corresponding to comparison of spine intensity across regions/layers with Sidak’s multiple comparisons test (Fig. 2-1a)

| ANOVA table | SS | DF | MS | F (DFn, DFd) | P value |
| --- | --- | --- | --- | --- | --- |
| Treatment (between columns) | 768984 | 2 | 384492 | F (2, 282) = 13.70 | P<0.0001 |
| Residual (within columns) | 7912773 | 282 | 28059 |  |  |
| Total | 8681757 | 284 |  |  |  |

| Sidak's multiple comparisons test | Mean Diff. | 95.00% CI of diff. | Summary | Adjusted P Value |
| --- | --- | --- | --- | --- |
| L5V vs. L5M | 96.85 | 40.05 to 153.6 | *** | 0.0002 |
| L5V vs. L2/3V | -24.22 | -83.24 to 34.81 | ns | 0.6927 |
| L5M vs. L2/3V | -121.1 | -181.0 to -61.09 | **** | <0.0001 |
