# Supplementary material for: AMPA Receptors Exist in Tunable Mobile and Immobile Synaptic Fractions In Vivo
Source: eNeuro. 2021 May 14;8(3):ENEURO.0015-21.2021. doi: 10.1523/ENEURO.0015-21.2021 (PMC8143022; doi:10.1523/ENEURO.0015-21.2021)
Supplement: Extended Data Figure 2-9 — 1-way ANOVA corresponding to comparison of shaft intensity across regions/layers with Sidak's multiple comparisons test (Fig. 2-1b). Download Figure 2-9, DOCX file. [file enu-eN-REV-0015-21-s14.docx]

Figure 2-9 | 1-way ANOVA corresponding to comparison of shaft intensity across regions/layers with Sidak’s multiple comparisons test (Fig. 2-1b)

| ANOVA table | SS | DF | MS | F (DFn, DFd) | P value |
| --- | --- | --- | --- | --- | --- |
| Treatment (between columns) | 315439 | 2 | 157719 | F (2, 282) = 5.673 | P=0.0038 |
| Residual (within columns) | 7840341 | 282 | 27803 |  |  |
| Total | 8155779 | 284 |  |  |  |

| Sidak's multiple comparisons test | Mean Diff. | 95.00% CI of diff. | Summary | Adjusted P Value |
| --- | --- | --- | --- | --- |
| L5V vs. L5M | -35.24 | -91.77 to 21.30 | ns | 0.3539 |
| L5V vs. L2/3V | -82.37 | -141.1 to -23.62 | ** | 0.0026 |
| L5M vs. L2/3V | -47.13 | -106.8 to 12.56 | ns | 0.1665 |
