# Supplementary material for: AMPA Receptors Exist in Tunable Mobile and Immobile Synaptic Fractions In Vivo
Source: eNeuro. 2021 May 14;8(3):ENEURO.0015-21.2021. doi: 10.1523/ENEURO.0015-21.2021 (PMC8143022; doi:10.1523/ENEURO.0015-21.2021)
Supplement: Extended Data Figure 2-10 — 1-way ANOVA corresponding to comparison of synapse nearest neighbor distance across regions/layers with Sidak's multiple comparisons test (Fig. 2-1c). Download Figure 2-10, DOCX file. [file enu-eN-REV-0015-21-s15.docx]

Figure 2-10 | 1-way ANOVA corresponding to comparison of synapse nearest neighbor distance across regions/layers with Sidak’s multiple comparisons test (Fig. 2-1c)

| ANOVA table | SS | DF | MS | F (DFn, DFd) | P value |
| --- | --- | --- | --- | --- | --- |
| Treatment (between columns) | 6.112 | 2 | 3.056 | F (2, 255) = 18.56 | P<0.0001 |
| Residual (within columns) | 41.98 | 255 | 0.1646 |  |  |
| Total | 48.09 | 257 |  |  |  |

| Sidak's multiple comparisons test | Mean Diff. | 95.00% CI of diff. | Summary | Adjusted P Value |
| --- | --- | --- | --- | --- |
| L5V vs. L5M | 0.3636 | 0.2173 to 0.5099 | **** | <0.0001 |
| L5V vs. L2/3V | 0.2396 | 0.09093 to 0.3884 | *** | 0.0004 |
| L5M vs. L2/3V | -0.1240 | -0.2759 to 0.02795 | ns | 0.1451 |
