# Supplementary material for: AMPA Receptors Exist in Tunable Mobile and Immobile Synaptic Fractions In Vivo
Source: eNeuro. 2021 May 14;8(3):ENEURO.0015-21.2021. doi: 10.1523/ENEURO.0015-21.2021 (PMC8143022; doi:10.1523/ENEURO.0015-21.2021)
Supplement: Extended Data Figure 2-11 — Exponential curve fit for fluorescence recovery across spine sizes (Fig. 2-1d). Download Figure 2-11, DOCX file. [file enu-eN-REV-0015-21-s17.docx]

Figure 2-11 | Exponential curve fit for fluorescence recovery across spine sizes (Fig. 2-1d)

|  | Small | Medium | Large |
| --- | --- | --- | --- |
| Best-fit values |  |  |  |
| YM | 0.4183 | 0.5042 | 0.5818 |
| Y0 | 0.02100 | 0.01793 | 0.02788 |
| k | 0.2679 | 0.1968 | 0.1005 |
| 95% CI (profile likelihood) |  |  |  |
| YM | 0.3819 to 0.4656 | 0.4635 to 0.5517 | 0.5295 to 0.6543 |
| Y0 | -0.03211 to 0.07243 | -0.03215 to 0.06669 | -0.01038 to 0.06550 |
| k | 0.1458 to 0.4997 | 0.1321 to 0.2979 | 0.07115 to 0.1364 |
| Goodness of Fit |  |  |  |
| Degrees of Freedom | 535 | 515 | 515 |
| R squared | 0.2987 | 0.3515 | 0.4623 |
| Sum of Squares | 28.60 | 32.81 | 23.57 |
| Sy.x | 0.2312 | 0.2524 | 0.2139 |
