# Supplementary material for: AMPA Receptors Exist in Tunable Mobile and Immobile Synaptic Fractions In Vivo
Source: eNeuro. 2021 May 14;8(3):ENEURO.0015-21.2021. doi: 10.1523/ENEURO.0015-21.2021 (PMC8143022; doi:10.1523/ENEURO.0015-21.2021)
Supplement: Extended Data Figure 2-12 — Multifactorial ANOVA corresponding to comparison of fluorescence recovery across spine sizes (Fig. 2-1d). Download Figure 2-12, DOCX file. [file enu-eN-REV-0015-21-s16.docx]

Figure 2-12 | Multifactorial ANOVA corresponding to comparison of fluorescence recovery across spine sizes (Fig. 2-1d)

| Fixed effects (type III) | P value | P value summary | F (DFn, DFd) |
| --- | --- | --- | --- |
| Time | <0.0001 | **** | F (4.149, 1057) = 298.5 |
| Column Factor | 0.3052 | ns | F (2, 282) = 1.192 |
| Time x Column Factor | 0.0011 | ** | F (10, 1274) = 2.963 |

| Sidak's multiple comparisons test | Mean Diff. | 95.00% CI of diff. | Summary | Adjusted P Value |
| --- | --- | --- | --- | --- |
| 1 vs. 2 | 0.07116 | -0.05247 to 0.1948 | ns | 0.4263 |
| 1 vs. 3 | 0.1674 | 0.04375 to 0.2910 | ** | 0.0037 |
| 2 vs. 3 | 0.09622 | -0.02858 to 0.2210 | ns | 0.1840 |
