# Supplementary material for: AMPA Receptors Exist in Tunable Mobile and Immobile Synaptic Fractions In Vivo
Source: eNeuro. 2021 May 14;8(3):ENEURO.0015-21.2021. doi: 10.1523/ENEURO.0015-21.2021 (PMC8143022; doi:10.1523/ENEURO.0015-21.2021)
Supplement: Extended Data Figure 2-13 — 1-way ANOVA corresponding to comparison of FRAP recovery rate across spine sizes with Sidak's multiple comparisons test (Fig. 2-1e). Download Figure 2-13, DOCX file. [file enu-eN-REV-0015-21-s18.docx]

Figure 2-13 | 1-way ANOVA corresponding to comparison of FRAP recovery rate across spine sizes with Sidak’s multiple comparisons test (Fig. 2-1e)

| ANOVA table | SS | DF | MS | F (DFn, DFd) | P value |
| --- | --- | --- | --- | --- | --- |
| Treatment (between columns) | 7.407 | 2 | 3.703 | F (2, 1565) = 5.266 | P=0.0053 |
| Residual (within columns) | 1101 | 1565 | 0.7033 |  |  |
| Total | 1108 | 1567 |  |  |  |
