# Supplementary material for: AMPA Receptors Exist in Tunable Mobile and Immobile Synaptic Fractions In Vivo
Source: eNeuro. 2021 May 14;8(3):ENEURO.0015-21.2021. doi: 10.1523/ENEURO.0015-21.2021 (PMC8143022; doi:10.1523/ENEURO.0015-21.2021)
Supplement: Extended Data Figure 3-3 — Exponential curve fit for fluorescence recovery in baseline, corticosterone-, or saline- treated mice (Fig. 3d, 3f). Download Figure 3-3, DOCX file. [file enu-eN-REV-0015-21-s21.docx]

Figure 3-3 | Exponential curve fit for fluorescence recovery in baseline, corticosterone-, or saline- treated mice (Fig. 3d, 3f)

|  | Baseline | Cort 1hr | Cort 2hr | Cort 3hr | Saline 1hr | Saline 2hr | Saline 3hr |
| --- | --- | --- | --- | --- | --- | --- | --- |
| Best-fit values |  |  |  |  |  |  |  |
| YM | 0.6153 | 0.5597 | 0.6359 | 0.9033 | 0.5753 | 0.6950 | 0.6448 |
| Y0 | 0.007214 | 0.01569 | -0.04018 | 0.06184 | -0.04801 | -0.01161 | 0.03225 |
| k | 0.1838 | 0.1367 | 0.2235 | 0.1217 | 0.3214 | 0.09682 | 0.1688 |
| 95% CI (profile likelihood) |  |  |  |  |  |  |  |
| YM | 0.5586 to 0.6833 | 0.4857 to 0.6690 | 0.5889 to 0.6875 | 0.8038 to 1.047 | 0.5047 to 0.6512 | 0.5773 to 0.9383 | 0.5746 to 0.7334 |
| Y0 | -0.05387 to 0.06423 | -0.05238 to 0.07949 | -0.09821 to 0.01570 | -0.01804 to 0.1382 | -0.1545 to 0.05451 | -0.08846 to 0.06207 | -0.03916 to 0.09701 |
| k | 0.1203 to 0.2839 | 0.07505 to 0.2436 | 0.1628 to 0.3048 | 0.07469 to 0.1913 | 0.1873 to 0.5162 | 0.04644 to 0.1703 | 0.1012 to 0.2952 |
| Goodness of Fit |  |  |  |  |  |  |  |
| Degrees of Freedom | 682 | 275 | 350 | 331 | 276 | 313 | 323 |
| R squared | 0.2998 | 0.3666 | 0.5259 | 0.4328 | 0.2674 | 0.3329 | 0.4038 |
| Sum of Squares | 88.19 | 20.63 | 21.90 | 43.46 | 44.43 | 41.04 | 27.08 |
| Sy.x | 0.3596 | 0.2739 | 0.2501 | 0.3623 | 0.4012 | 0.3621 | 0.2895 |
