# Supplementary material for: AMPA Receptors Exist in Tunable Mobile and Immobile Synaptic Fractions In Vivo
Source: eNeuro. 2021 May 14;8(3):ENEURO.0015-21.2021. doi: 10.1523/ENEURO.0015-21.2021 (PMC8143022; doi:10.1523/ENEURO.0015-21.2021)
Supplement: Extended Data Figure 3-4 — Multifactorial ANOVA corresponding to comparison of fluorescence recovery between baseline and times after saline injection and Sidak's multiple comparisons (Fig. 3d). Download Figure 3-4, DOCX file. [file enu-eN-REV-0015-21-s22.docx]

Figure 3-4 | Multifactorial ANOVA corresponding to comparison of fluorescence recovery between baseline and times after saline injection and Sidak’s multiple comparisons (Fig. 3d)

| Fixed effects (type III) | P value | P value summary | F (DFn, DFd) |
| --- | --- | --- | --- |
| Time | <0.0001 | **** | F (4.632, 1024) = 168.5 |
| Hour post injection | 0.5360 | ns | F (3, 251) = 0.7282 |
| Time x Hour post injection | 0.05 | ns | F (18, 1327) = 1.623 |
|  |  |  |  |
| Random effects | SD | Variance |  |
| Subject | 0.2144 | 0.04597 |  |
| Residual | 0.2841 | 0.08073 |  |

| Sidak's multiple comparisons test | Mean Diff. | 95.00% CI of diff. | Summary | Adjusted P Value |
| --- | --- | --- | --- | --- |
| Row 2 |  |  |  |  |
| Baseline vs. Saline 1hr | 0.05323 | -0.1129 to 0.2193 | ns | 0.9459 |
| Baseline vs. Saline 2hr | 0.08978 | -0.06276 to 0.2423 | ns | 0.5200 |
| Baseline vs. Saline 3hr | -0.01661 | -0.1354 to 0.1022 | ns | 0.9994 |
| Saline 1hr vs. Saline 2hr | 0.03655 | -0.1683 to 0.2414 | ns | 0.9975 |
| Saline 1hr vs. Saline 3hr | -0.06984 | -0.2527 to 0.1130 | ns | 0.8869 |
| Saline 2hr vs. Saline 3hr | -0.1064 | -0.2774 to 0.06460 | ns | 0.4593 |
| Row 3 |  |  |  |  |
| Baseline vs. Saline 1hr | -0.01774 | -0.2394 to 0.2039 | ns | >0.9999 |
| Baseline vs. Saline 2hr | 0.09079 | -0.1162 to 0.2978 | ns | 0.8058 |
| Baseline vs. Saline 3hr | -0.07503 | -0.2132 to 0.06315 | ns | 0.6194 |
| Saline 1hr vs. Saline 2hr | 0.1085 | -0.1625 to 0.3796 | ns | 0.8649 |
| Saline 1hr vs. Saline 3hr | -0.05729 | -0.2839 to 0.1693 | ns | 0.9832 |
| Saline 2hr vs. Saline 3hr | -0.1658 | -0.3782 to 0.04657 | ns | 0.2082 |
| Row 4 |  |  |  |  |
| Baseline vs. Saline 1hr | -0.1144 | -0.3461 to 0.1173 | ns | 0.7039 |
| Baseline vs. Saline 2hr | 0.07751 | -0.09791 to 0.2529 | ns | 0.8030 |
| Baseline vs. Saline 3hr | 0.06047 | -0.09765 to 0.2186 | ns | 0.8893 |
| Saline 1hr vs. Saline 2hr | 0.1919 | -0.06158 to 0.4454 | ns | 0.2372 |
| Saline 1hr vs. Saline 3hr | 0.1749 | -0.06795 to 0.4177 | ns | 0.2873 |
| Saline 2hr vs. Saline 3hr | -0.01704 | -0.2078 to 0.1737 | ns | >0.9999 |
| Row 5 |  |  |  |  |
| Baseline vs. Saline 1hr | -0.07277 | -0.2924 to 0.1469 | ns | 0.9385 |
| Baseline vs. Saline 2hr | 0.05906 | -0.1338 to 0.2519 | ns | 0.9587 |
| Baseline vs. Saline 3hr | -0.06090 | -0.2442 to 0.1224 | ns | 0.9402 |
| Saline 1hr vs. Saline 2hr | 0.1318 | -0.1114 to 0.3751 | ns | 0.6147 |
| Saline 1hr vs. Saline 3hr | 0.01187 | -0.2244 to 0.2481 | ns | >0.9999 |
| Saline 2hr vs. Saline 3hr | -0.1200 | -0.3322 to 0.09226 | ns | 0.5711 |
| Row 6 |  |  |  |  |
| Baseline vs. Saline 1hr | 0.09105 | -0.1252 to 0.3073 | ns | 0.8350 |
| Baseline vs. Saline 2hr | 0.02691 | -0.1835 to 0.2373 | ns | 0.9996 |
| Baseline vs. Saline 3hr | -0.01285 | -0.1917 to 0.1660 | ns | >0.9999 |
| Saline 1hr vs. Saline 2hr | -0.06414 | -0.3035 to 0.1753 | ns | 0.9782 |
| Saline 1hr vs. Saline 3hr | -0.1039 | -0.3174 to 0.1096 | ns | 0.7214 |
| Saline 2hr vs. Saline 3hr | -0.03976 | -0.2473 to 0.1678 | ns | 0.9963 |
| Row 7 |  |  |  |  |
| Baseline vs. Saline 1hr | 0.05431 | -0.1966 to 0.3052 | ns | 0.9928 |
| Baseline vs. Saline 2hr | -0.04979 | -0.2658 to 0.1662 | ns | 0.9902 |
| Baseline vs. Saline 3hr | -0.02897 | -0.2318 to 0.1739 | ns | 0.9993 |
| Saline 1hr vs. Saline 2hr | -0.1041 | -0.3709 to 0.1627 | ns | 0.8777 |
| Saline 1hr vs. Saline 3hr | -0.08328 | -0.3400 to 0.1735 | ns | 0.9450 |
| Saline 2hr vs. Saline 3hr | 0.02082 | -0.2024 to 0.2441 | ns | >0.9999 |
