# Supplementary material for: AMPA Receptors Exist in Tunable Mobile and Immobile Synaptic Fractions In Vivo
Source: eNeuro. 2021 May 14;8(3):ENEURO.0015-21.2021. doi: 10.1523/ENEURO.0015-21.2021 (PMC8143022; doi:10.1523/ENEURO.0015-21.2021)
Supplement: Extended Data Figure 3-5 — 1-way ANOVA corresponding to comparison of mobile fraction baseline and times after saline injection (Fig. 3e). Download Figure 3-5, DOCX file. [file enu-eN-REV-0015-21-s23.docx]

Figure 3-5 | 1-way ANOVA corresponding to comparison of mobile fraction baseline and times after saline injection (Fig. 3e)

| ANOVA table | SS | DF | MS | F (DFn, DFd) | P value |
| --- | --- | --- | --- | --- | --- |
| Treatment (between columns) | 2.377 | 3 | 0.7922 | F (3, 1594) = 1.129 | P=0.3359 |
| Residual (within columns) | 1118 | 1594 | 0.7014 |  |  |
| Total | 1120 | 1597 |  |  |  |
