# Supplementary material for: AMPA Receptors Exist in Tunable Mobile and Immobile Synaptic Fractions In Vivo
Source: eNeuro. 2021 May 14;8(3):ENEURO.0015-21.2021. doi: 10.1523/ENEURO.0015-21.2021 (PMC8143022; doi:10.1523/ENEURO.0015-21.2021)
Supplement: Extended Data Figure 3-6 — Multifactorial ANOVA corresponding to comparison of fluorescence recovery between baseline and times after corticosterone injection and Sidak's multiple comparisons (Fig. 3f). Download Figure 3-6, DOCX file. [file enu-eN-REV-0015-21-s24.docx]

Figure 3-6 | Multifactorial ANOVA corresponding to comparison of fluorescence recovery between baseline and times after corticosterone injection and Sidak’s multiple comparisons (Fig. 3f)

| Fixed effects (type III) | P value | P value summary | F (DFn, DFd) |
| --- | --- | --- | --- |
| Time | <0.0001 | **** | F (4.331, 989.7) = 234.4 |
| Hour post injection | 0.0002 | *** | F (3, 251) = 6.709 |
| Time x Hour post injection | 0.0002 | *** | F (18, 1371) = 2.642 |
|  |  |  |  |
| Random effects | SD | Variance |  |
| Subject | 0.1921 | 0.03691 |  |
| Residual | 0.2634 | 0.06939 |  |

| Sidak's multiple comparisons | Mean Diff. | 95.00% CI of diff. | Summary | Adjusted P Value |
| --- | --- | --- | --- | --- |
| Row 2 |  |  |  |  |
| Baseline vs. Cort 1hr | 0.02466 | -0.1066 to 0.1559 | ns | 0.9966 |
| Baseline vs. Cort 2hr | 0.08978 | -0.009440 to 0.1890 | ns | 0.0976 |
| Baseline vs. Cort 3hr | -0.09676 | -0.2397 to 0.04614 | ns | 0.3576 |
| Cort 1hr vs. Cort 2hr | 0.06512 | -0.07366 to 0.2039 | ns | 0.7539 |
| Cort 1hr vs. Cort 3hr | -0.1214 | -0.2927 to 0.04986 | ns | 0.3084 |
| Cort 2hr vs. Cort 3hr | -0.1865 | -0.3364 to -0.03672 | ** | 0.0072 |
| Row 3 |  |  |  |  |
| Baseline vs. Cort 1hr | 0.03225 | -0.09788 to 0.1624 | ns | 0.9858 |
| Baseline vs. Cort 2hr | -0.01757 | -0.1386 to 0.1034 | ns | 0.9993 |
| Baseline vs. Cort 3hr | -0.08641 | -0.2542 to 0.08137 | ns | 0.6703 |
| Cort 1hr vs. Cort 2hr | -0.04982 | -0.1726 to 0.07298 | ns | 0.8572 |
| Cort 1hr vs. Cort 3hr | -0.1187 | -0.2873 to 0.04997 | ns | 0.3147 |
| Cort 2hr vs. Cort 3hr | -0.06884 | -0.2310 to 0.09337 | ns | 0.8285 |
| Row 4 |  |  |  |  |
| Baseline vs. Cort 1hr | 0.1145 | -0.02476 to 0.2537 | ns | 0.1654 |
| Baseline vs. Cort 2hr | -0.05220 | -0.1956 to 0.09122 | ns | 0.9116 |
| Baseline vs. Cort 3hr | -0.05625 | -0.2180 to 0.1055 | ns | 0.9266 |
| Cort 1hr vs. Cort 2hr | -0.1667 | -0.3112 to -0.02213 | * | 0.0153 |
| Cort 1hr vs. Cort 3hr | -0.1707 | -0.3332 to -0.008189 | * | 0.0344 |
| Cort 2hr vs. Cort 3hr | -0.004053 | -0.1702 to 0.1620 | ns | >0.9999 |
| Row 5 |  |  |  |  |
| Baseline vs. Cort 1hr | 0.06220 | -0.1212 to 0.2456 | ns | 0.9337 |
| Baseline vs. Cort 2hr | -0.06415 | -0.2220 to 0.09368 | ns | 0.8600 |
| Baseline vs. Cort 3hr | -0.2021 | -0.4019 to -0.002353 | * | 0.0459 |
| Cort 1hr vs. Cort 2hr | -0.1264 | -0.3084 to 0.05564 | ns | 0.3300 |
| Cort 1hr vs. Cort 3hr | -0.2643 | -0.4827 to -0.04598 | ** | 0.0096 |
| Cort 2hr vs. Cort 3hr | -0.1380 | -0.3364 to 0.06046 | ns | 0.3291 |
| Row 6 |  |  |  |  |
| Baseline vs. Cort 1hr | 0.08306 | -0.1141 to 0.2802 | ns | 0.8367 |
| Baseline vs. Cort 2hr | -0.04057 | -0.2134 to 0.1322 | ns | 0.9894 |
| Baseline vs. Cort 3hr | -0.2033 | -0.4100 to 0.003445 | ns | 0.0564 |
| Cort 1hr vs. Cort 2hr | -0.1236 | -0.3125 to 0.06521 | ns | 0.3978 |
| Cort 1hr vs. Cort 3hr | -0.2863 | -0.5059 to -0.06675 | ** | 0.0043 |
| Cort 2hr vs. Cort 3hr | -0.1627 | -0.3615 to 0.03604 | ns | 0.1676 |
| Row 7 |  |  |  |  |
| Baseline vs. Cort 1hr | 0.07147 | -0.1326 to 0.2755 | ns | 0.9242 |
| Baseline vs. Cort 2hr | 0.01139 | -0.1689 to 0.1917 | ns | >0.9999 |
| Baseline vs. Cort 3hr | -0.2817 | -0.5184 to -0.04499 | * | 0.0113 |
| Cort 1hr vs. Cort 2hr | -0.06007 | -0.2510 to 0.1309 | ns | 0.9524 |
| Cort 1hr vs. Cort 3hr | -0.3532 | -0.5972 to -0.1091 | ** | 0.0012 |
| Cort 2hr vs. Cort 3hr | -0.2931 | -0.5186 to -0.06754 | ** | 0.0045 |
