# Supplementary material for: AMPA Receptors Exist in Tunable Mobile and Immobile Synaptic Fractions In Vivo
Source: eNeuro. 2021 May 14;8(3):ENEURO.0015-21.2021. doi: 10.1523/ENEURO.0015-21.2021 (PMC8143022; doi:10.1523/ENEURO.0015-21.2021)
Supplement: Extended Data Figure 3-7 — 1-way ANOVA and Sidak's multiple comparisons test corresponding to comparison of mobile fraction baseline and times after corticosterone injection (Fig. 3g). Download Figure 3-7, DOCX file. [file enu-eN-REV-0015-21-s25.docx]

Figure 3-7 | 1-way ANOVA and Sidak’s multiple comparisons test corresponding to comparison of mobile fraction baseline and times after corticosterone injection (Fig. 3g)

| ANOVA table | SS | DF | MS | F (DFn, DFd) | P value |
| --- | --- | --- | --- | --- | --- |
| Treatment (between columns) | 23.87 | 3 | 7.956 | F (3, 1638) = 14.54 | P<0.0001 |
| Residual (within columns) | 896.0 | 1638 | 0.5470 |  |  |
| Total | 919.9 | 1641 |  |  |  |

| Sidak's multiple comparisons | Mean Diff. | 95.00% CI of diff. | Summary | Adjusted P Value |
| --- | --- | --- | --- | --- |
| Baseline vs. Cort 1hr | 0.05558 | -0.08338 to 0.1945 | ns | 0.8743 |
| Baseline vs. Cort 2hr | -0.02059 | -0.1485 to 0.1074 | ns | 0.9987 |
| Baseline vs. Cort 3hr | -0.2879 | -0.4183 to -0.1576 | **** | <0.0001 |
| Cort 1hr vs. Cort 2hr | -0.07617 | -0.2329 to 0.08057 | ns | 0.7391 |
| Cort 1hr vs. Cort 3hr | -0.3435 | -0.5022 to -0.1848 | **** | <0.0001 |
| Cort 2hr vs. Cort 3hr | -0.2673 | -0.4165 to -0.1182 | **** | <0.0001 |
