# Supplementary material for: AMPA Receptors Exist in Tunable Mobile and Immobile Synaptic Fractions In Vivo
Source: eNeuro. 2021 May 14;8(3):ENEURO.0015-21.2021. doi: 10.1523/ENEURO.0015-21.2021 (PMC8143022; doi:10.1523/ENEURO.0015-21.2021)
Supplement: Extended Data Figure 3-8 — 2-way ANOVA and Sidak's multiple comparison test corresponding to comparison between fluorescence recovery at 32 min for corticosterone vs saline injection at 1-, 2-, and 3-hours post-injection (Fig. 3h). Download Figure 3-8, DOCX file. [file enu-eN-REV-0015-21-s26.docx]

Figure 3-8 | 2-way ANOVA and Sidak’s multiple comparison test corresponding to comparison between fluorescence recovery at 32 min for corticosterone vs saline injection at 1-, 2-, and 3-hours post-injection (Fig. 3h)

| Two-way ANOVA | Ordinary |  |  |  |  |
| --- | --- | --- | --- | --- | --- |
| Alpha | 0.05 |  |  |  |  |
|  |  |  |  |  |  |
| Source of Variation | % of total variation | P value | P value summary | Significant? |  |
| Interaction | 2.793 | 0.0220 | * | Yes |  |
| Hour | 4.508 | 0.0022 | ** | Yes |  |
| Treatment | 0.4785 | 0.2502 | ns | No |  |
|  |  |  |  |  |  |
| ANOVA table | SS (Type III) | DF | MS | F (DFn, DFd) | P value |
| Interaction | 1.280 | 2 | 0.6400 | F (2, 255) = 3.876 | P=0.0220 |
| Hour | 2.066 | 2 | 1.033 | F (2, 255) = 6.257 | P=0.0022 |
| Treatment | 0.2193 | 1 | 0.2193 | F (1, 255) = 1.328 | P=0.2502 |
| Residual | 42.10 | 255 | 0.1651 |  |  |

| Sidak's multiple comparisons | Predicted (LS) mean diff. | 95.00% CI of diff. | Summary | Adjusted P Value |
| --- | --- | --- | --- | --- |
| Cort - Saline |  |  |  |  |
| 1 | -0.01716 | -0.2356 to 0.2013 | ns | 0.9967 |
| 2 | -0.06118 | -0.2650 to 0.1426 | ns | 0.8522 |
| 3 | 0.2527 | 0.04536 to 0.4601 | * | 0.0111 |
